# Early SOX9 Activation Primes Hippo–YAP/TAZ Rewiring During Glioblastoma Stemness Acquisition

**DOI:** 10.1111/jcmm.71341

**Published:** 2026-08-30

**Authors:** Maimaitili Mijiti, Yandong Li, Aierpati Maimaiti, Gaocai Zhang, Minghao Lian, Chunyu Song, Yongtao Zhang, Jufan Wang, Yunbo Wang, Xiaoqin Chen, Xiangrui Kong, Xiahela Xiaokaiti, Nuerzhati Wusiman, Guohua Zhu, Dangmurenjiafu Geng, Hua Shi

**Affiliations:** ^1^ Neurosurgery Centre, Department of Neurosurgery Xinjiang Medical University Affiliated First Hospital Urumqi Xinjiang China; ^2^ Department of Neurosurgery Shandong Second Medical University Affiliated Linyi People's Hospital Linyi Shandong China

**Keywords:** glioblastoma, hippo signalling, SOX9, spatial transcriptomics, stemness, YAP/TAZ

## Abstract

Glioblastoma progression is driven by stemness acquisition and cellular plasticity, yet the temporal and spatial organization of these processes remains incompletely defined. In this study, single‐cell RNA sequencing, pseudotime reconstruction, spatial transcriptomics, and in vitro and in vivo functional assays were integrated to delineate the role of SOX9 during glioblastoma stemness acquisition. SOX9 expression peaked during the early astrocyte‐to‐malignant transition and declined after malignant states became stabilized. Along pseudotime, SOX9 was inversely associated with the upstream Hippo kinase module and showed phase‐dependent coupling with YAP/TAZ‐associated transcriptional programmes. Spatial analyses further revealed marked regional heterogeneity, with the strongest SOX9‐malignant coupling observed in the perivascular niche. Functionally, SOX9 gain and loss produced reciprocal changes in glioblastoma cell proliferation, migration, invasion, apoptosis, and xenograft growth, while pharmacological modulation of Hippo signalling partially rescued the effects induced by SOX9 loss. Consistent pathway‐level changes were also observed in YAP/TAZ expression and p‐YAP/YAP and p‐MOB1/MOB1 ratios, and xenograft histology showed SOX9‐associated morphological and CD68‐positive cell changes. These findings identify SOX9 as a temporally restricted regulator of glioblastoma stemness acquisition and support an early priming‐late decoupling model of Hippo‐YAP/TAZ rewiring, providing a rationale for stage‐specific and niche‐aware therapeutic targeting in glioblastoma.

**Abbreviations**
AOD: average optical density
ATCC: American Type Culture Collection
AUCell: Area Under the Curve cell‐level scoring method
BCA: bicinchoninic acid
BSA: bovine serum albumin
cDNA: complementary DNA
CRISPR: clustered regularly interspaced short palindromic repeats
DAB: 3,3′‐diaminobenzidine
DMEM: Dulbecco's modified Eagle's medium
ECL: enhanced chemiluminescence
FBS: foetal bovine serum
GAM: generalized additive model
GAPDH: glyceraldehyde‐3‐phosphate dehydrogenase
GBM: glioblastoma
GEO: Gene Expression Omnibus
GSVA: Gene Set Variation Analysis
H&E: haematoxylin and eosin
HRP: horseradish peroxidase
KO: knockout
MEM: minimum essential medium
NC: negative control
OE: overexpression
PBS: phosphate‐buffered saline
PC: principal component
PCA: principal component analysis
PI: propidium iodide
PS: penicillin–streptomycin
PVDF: polyvinylidene fluoride
qRT‐PCR: quantitative real‐time polymerase chain reaction
RIPA: radioimmunoprecipitation assay
RNA‐seq: RNA sequencing
SCTransform: regularized negative binomial regression normalization
SDS‐PAGE: sodium dodecyl sulphate polyacrylamide gel electrophoresis
sgRNA: single‐guide RNA
SNN: shared nearest neighbour
SPF: specific pathogen‐free
STR: short tandem repeat
TEAD: TEA domain transcription factor
TRIzol: guanidinium thiocyanate–phenol–chloroform RNA isolation reagent
UMAP: uniform manifold approximation and projection
VST: variance‐stabilizing transformation

## 1 Introduction

Glioblastoma (GBM) progression is driven in part by the acquisition of stem‐like traits and phenotypic plasticity during malignant evolution [1, 2]. Rather than representing a fixed cellular entity, the glioma stem cell (GSC) state is increasingly recognized as a dynamic and context‐dependent phenotype. Among the pathways implicated in this process, Hippo signalling and its downstream effectors YAP/TAZ are key regulators of stemness‐associated transcriptional programmes [3, 4]. However, the upstream events that contribute to the temporal engagement of Hippo–YAP/TAZ signalling during stemness acquisition remain poorly defined.

SOX9 is a developmental transcription factor involved in neural lineage specification and glioma biology [5]. Previous studies have linked SOX9 to glioma stem cell maintenance and treatment resistance [6], but most have relied on bulk or static analyses, leaving unresolved whether SOX9 acts as a constitutive stemness factor or as a temporally restricted regulator during malignant state transition. In parallel, stemness‐related programmes in GBM are spatially enriched in specific microenvironmental niches, particularly the perivascular compartment [7, 8], yet whether SOX9‐associated Hippo–YAP/TAZ pathway engagement also shows spatial specificity remains unclear.

To address these questions, single‐cell trajectory analysis, spatial transcriptomics, in vitro and in vivo functional assays, phosphorylation‐level pathway assessment, and histological validation were integrated to define the temporal and spatial role of SOX9 in GBM. The study aimed to determine whether SOX9 functions as a temporally restricted regulator associated with Hippo–YAP/TAZ‐linked malignant reprogramming during glioma progression.

## 2 Materials and Methods

### 2.1 Single‐Cell Transcriptomic Analysis

#### 2.1.1 Data Acquisition, Preprocessing, and Quality Control

Single‐cell RNA sequencing data were obtained from the Gene Expression Omnibus (GEO) under accession number GSE182109. The dataset was generated using the 10× Genomics Chromium platform and processed in R using the Seurat package. Raw gene–barcode count matrices were imported as sparse matrices. Cells with fewer than 50 detected genes or with mitochondrial transcript proportions > 5% were excluded. Gene expression was normalized using the LogNormalize method with a scaling factor of 10,000, and 1500 highly variable genes were identified using the variance‐stabilizing transformation method. When multiple samples were included, Seurat integration was performed before downstream analyses to reduce batch effects.

#### 2.1.2 Dimensionality Reduction, Clustering, and Cell Type Annotation

Principal component analysis (PCA) was performed using the highly variable genes, and the top 20 principal components were selected for downstream analysis based on JackStraw testing. A shared nearest neighbour graph was constructed, followed by Louvain clustering at a resolution of 0.5. Cellular distributions were visualized using uniform manifold approximation and projection (UMAP).

Cell type annotation was performed using SingleR with multiple human reference datasets. Annotations were further refined according to canonical marker gene expression to ensure biological consistency.

#### 2.1.3 Pseudotime Trajectory Reconstruction and Dynamic Gene Analysis

Pseudotime analysis was performed using Monocle3 to reconstruct the transition trajectory from astrocyte‐like states to malignant cell states. Root cells were assigned to astrocyte‐dominant early states based on cluster identity and marker expression. To characterize local trajectory structure, a *k*‐nearest neighbour approach (*k* = 50) was used to define neighbourhood‐level nodes, from which dominant cell type, mean pseudotime, and gene expression profiles were summarized.

Genes associated with pseudotime progression were identified using the graph_test function in Monocle3. The top pseudotime‐associated genes were visualized in heatmaps after *Z*‐score normalization to illustrate dynamic transcriptional changes along the trajectory.

#### 2.1.4 Pathway Activity Estimation and Nonlinear Modelling

Pathway activity was estimated using Gene Set Variation Analysis (GSVA), and AUCell and AddModuleScore were used as complementary approaches for robustness assessment. The upstream Hippo kinase module included MST1/2, LATS1/2, SAV1, MOB1A/B, NF2, and WWC1, and the YAP/TAZ target module included CTGF, CYR61, ANKRD1, AMOTL2, AREG, SERPINE1, FOSL1, AXL, ITGA6, and BIRC5 [4, 9]. Pathway scores were integrated with pseudotime values for downstream analysis.

Global associations were assessed using Pearson or Spearman correlation as appropriate. Nonlinear relationships between pseudotime, SOX9 expression, and pathway activity were modelled using generalized additive models implemented in the mgcv package.

### 2.2 Spatial Transcriptomic Analysis

#### 2.2.1 Data Preprocessing and Cross‐Platform Annotation

Spatial transcriptomic data were obtained from GEO under accession number GSE253080. The dataset was generated using the 10× Genomics Visium platform and processed in Seurat. Tissue regions were independently annotated by senior board‐certified pathologists and classified as tumour–normal interface, pure tumour, perivascular, or tumour–necrosis interface. Raw spatial data were normalized using SCTransform, and 3000 variable features were identified. The top 30 principal components were used for downstream analyses.

For cross‐platform annotation, the single‐cell transcriptomic dataset was used as the reference. Transfer anchors were identified using FindTransferAnchors, and cell type probabilities for each spatial spot were generated using TransferData. Malignant cell probabilities were extracted for subsequent analyses.

#### 2.2.2 Spatial Expression Mapping and Spot Classification

Normalized SOX9 expression and malignant cell probability were mapped onto tissue sections using spatial feature visualization. For point pattern and neighbourhood analyses, SOX9‐high spots were defined using the upper quartile of SOX9 expression, whereas malignant‐high spots were defined as spots with malignant cell probabilities > 0.5. Spatial coordinates were integrated with expression values, malignant cell probabilities, and spot labels for downstream co‐localization analyses.

#### 2.2.3 Spatial Co‐Localization and Point Pattern Analysis

The association between SOX9 expression and malignant cell probability was first evaluated using Pearson correlation. Spatial point pattern analysis was then performed by converting SOX9‐high and malignant‐high spots into multitype point patterns and estimating cross‐type spatial relationships using the Kcross function.

Neighbourhood enrichment analysis was performed by constructing spatial adjacency graphs using a *k*‐nearest neighbour approach (*k* = 6). Interaction edges between SOX9‐high and malignant‐high spots were quantified, and enrichment significance was assessed using 999 permutation tests. Global spatial autocorrelation was further evaluated using bivariate Moran's *I* and Lee's *L* statistics with permutation‐based null distributions.

### 2.3 Cell Culture and Generation of Stable Cell Lines

#### 2.3.1 Cell Culture, Authentication, and Baseline SOX9 Expression Assessment

U‐87MG and U251 human glioma cell lines were obtained from the American Type Culture Collection (ATCC, Manassas, VA, USA). U87 cells were cultured in Minimum Essential Medium (MEM; Gibco, Grand Island, NY, USA) supplemented with 10% foetal bovine serum (FBS; Gibco) and 1% penicillin–streptomycin (Gibco). U251 cells were maintained in Dulbecco's Modified Eagle Medium (DMEM; Gibco) supplemented with 10% FBS and 1% penicillin–streptomycin. All cells, including the stable cell lines established in this study, were cultured at 37°C in a humidified incubator with 5% CO_2_.

Cell line identity was verified by short tandem repeat (STR) profiling using an ABI 3730xl DNA Analyzer (Applied Biosystems, Foster City, CA, USA). STR profiles were analyzed using GeneMapper ID‐X software (v1.6; Applied Biosystems) and compared with reference databases. All cell lines were routinely tested and confirmed to be free of mycoplasma contamination before experimentation.

Baseline SOX9 expression was assessed by quantitative real‐time PCR (qRT‐PCR). Total RNA was extracted using TRIzol reagent (Invitrogen, Carlsbad, CA, USA), and RNA concentration and purity were determined using a NanoDrop 2000 spectrophotometer (Thermo Fisher Scientific, Waltham, MA, USA). One microgram of total RNA was reverse transcribed using the PrimeScript RT reagent kit (Takara Bio, Kusatsu, Japan). qRT‐PCR was performed using SYBR Premix Ex Taq (Takara) on an ABI Prism 7500 system (Applied Biosystems). Relative SOX9 expression was calculated using the $2^{-\Delta \Delta C_{t}}$ method with GAPDH as the internal control. Primer sequences were as follows: SOX9 forward, 5′‐CCCGCTCACAGTACGACTAC‐3′; SOX9 reverse, 5′‐CTGAGCGGGGTTCATGTAGG‐3′; GAPDH forward, 5′‐GTCTTCACCACCATGGAGAA‐3′; and GAPDH reverse, 5′‐TAAGCAGTTGGTGGTGCAG‐3′.

#### 2.3.2 Lentiviral SOX9 Overexpression and CRISPR/Cas9‐Mediated Knockout

For SOX9 overexpression, the full‐length human SOX9 coding sequence (GenBank: NM_000346.4) was cloned into the pcSLenti‐CMV‐3xFLAG‐PGK‐Puro‐WPRE3 vector (H37908). The corresponding empty vector (GL186) served as the negative control. Insert orientation and sequence integrity were verified by Sanger sequencing.

For SOX9 knockout, single‐guide RNAs (sgRNAs) targeting SOX9 were designed and cloned into a Lenti‐Cas9‐Blast vector. Recombinant plasmids were transformed into Stbl3 competent *Escherichia coli* (Thermo Fisher Scientific) and verified by Sanger sequencing. Lentiviral particles for overexpression and knockout were produced in HEK293T cells by co‐transfection with the packaging plasmids psPAX2 and pMD2.G using Lipofectamine 3000 (Invitrogen). Viral supernatants were collected at 48 and 72 h after transfection, passed through 0.45‐μm filters, and concentrated by ultracentrifugation.

#### 2.3.3 Establishment of Stable Cell Lines

For SOX9 overexpression, U87 cells were seeded in 6‐well plates at a density of 2 × 10^5^ cells/well and infected with lentiviral particles in the presence of Polybrene (5 μg/mL; Sigma‐Aldrich, St. Louis, MO, USA). After 72 h, puromycin (4 μg/mL; InvivoGen, San Diego, CA, USA) was added for selection for approximately 2 weeks, with medium replaced every 2–3 days.

For SOX9 knockout, U251 cells were infected with lentiviral particles carrying Cas9 and SOX9‐targeting sgRNA under the same selection conditions. After puromycin selection, single‐cell clones were isolated by limiting dilution in 96‐well plates. Genomic DNA was extracted from individual clones, and the target region was amplified by PCR followed by Sanger sequencing. Clone KO#4, which carried a 2‐bp deletion (c.121_122del) predicted to cause a frameshift and premature stop codon, was used for subsequent experiments.

#### 2.3.4 Validation of Stable Cell Lines and XMU‐MP‐1 Treatment

Stable SOX9 overexpression and knockout were validated by qRT‐PCR and Western blotting. For Western blotting, total protein was extracted using RIPA lysis buffer supplemented with protease inhibitors. For phosphorylation‐dependent Hippo pathway assessment, protein samples were prepared from six experimental groups: U87‐OE‐NC, U87‐OE‐SOX9, U87‐OE‐SOX9 + XMU‐MP‐1, U251‐KO‐NC, U251‐KO‐SOX9, and U251‐KO‐SOX9 + XMU‐MP‐1. Equal amounts of protein were separated by SDS‐PAGE and transferred onto PVDF membranes. Membranes were blocked and incubated overnight at 4°C with primary antibodies against SOX9 (bioss, bs‐4177R, 1:1000), YAP1 (Proteintech, 13584‐1‐AP, 1:2000), TAZ (Proteintech, 23306‐1‐AP, 1:1000), MOB1 (Proteintech, 11669‐1‐AP, 1:500), phosphorylated MOB1 (Proteintech, 29027‐1‐AP, 1:500), total YAP (Bioss, bsm‐52418R, 1:1000), phosphorylated YAP (Bioss, bsm‐52214R, 1:1000), and GAPDH (Proteintech, 60004‐1‐Ig, 1:50,000), followed by HRP‐conjugated secondary antibodies. Protein bands were detected using enhanced chemiluminescence. Band intensities were quantified, and the ratios of p‐MOB1/MOB1 and p‐YAP/YAP were calculated to evaluate phosphorylation‐dependent Hippo–YAP pathway changes. For in vitro pharmacological intervention, cells in the indicated groups were treated with XMU‐MP‐1 (MCE, HY‐100526) at 3 μM for 8 h, whereas control cells received vehicle treatment.

### 2.4 In Vitro Functional Assays

#### 2.4.1 Cell Proliferation Assay

Cell proliferation was assessed using a Cell Counting Kit‐8 (CCK‐8; Beyotime, Shanghai, China). Cells were seeded in 96‐well plates at a density of 1 × 10^3^ cells per well and cultured for 24 h. Cells in the indicated groups were then treated with XMU‐MP‐1 (MCE, HY‐100526) at a final concentration of 3 μM for 8 h, whereas control cells received vehicle treatment. Subsequently, 10 μL of CCK‐8 reagent was added to each well and incubated for 1 h at 37°C. Absorbance was measured at 450 nm using a microplate reader. Experiments were performed in triplicate and repeated at least three times.

#### 2.4.2 Wound‐Healing Assay

Cells were seeded in 6‐cm dishes at 5 × 10^5^ cells per dish and cultured for 24 h until reaching near confluence. A linear wound was created using a sterile 200‐μL pipette tip, and detached cells were removed by washing with PBS. Cells in the indicated groups were then treated with XMU‐MP‐1 (3 μM for 8 h), whereas control cells received vehicle treatment. Wound images were captured at the indicated time points, and wound closure was quantified using ImageJ software.

#### 2.4.3 Transwell Invasion Assay

Transwell invasion assays were performed using 24‐well Boyden chambers with Matrigel‐coated inserts (8‐μm pore size; Corning, NY, USA). Cells were resuspended in serum‐free medium and seeded into the upper chamber at a density of 1 × 10^3^ cells per well in 100 μL. Cells in the indicated groups were treated with XMU‐MP‐1 (3 μM for 8 h), whereas control cells received vehicle treatment. After 24 h incubation, cells remaining on the upper surface were removed, and cells that had invaded to the lower surface were fixed with 4% paraformaldehyde, stained with crystal violet, and counted under a microscope.

#### 2.4.4 Apoptosis Assay

Cell apoptosis was assessed using an Annexin V‐FITC/PI apoptosis detection kit (Beibo Bio, catalogue no. 401006) according to the manufacturer's instructions. Cells were seeded in 6‐well plates at 5 × 10^5^ cells/mL (2 mL per well) and cultured for 24 h. Cells in the indicated groups were then treated with XMU‐MP‐1 (3 μM for 8 h), whereas control cells received vehicle treatment. Cells were harvested using EDTA‐free trypsin, washed twice with cold PBS, stained with Annexin V‐FITC and propidium iodide, and analyzed by flow cytometry. The proportion of apoptotic cells was quantified.

### 2.5 In Vivo Tumour Formation Assay

Female BALB/c nude mice aged 4–6 weeks were maintained under specific pathogen‐free conditions. All animal procedures were approved by the institutional ethics committee and performed in accordance with relevant guidelines for animal care and use. Mice were assigned to six groups (*n* = 3 per group): U87‐OE‐NC, U87‐OE‐SOX9, U87‐OE‐SOX9 + XMU‐MP‐1, U251‐KO‐NC, U251‐KO‐SOX9, and U251‐KO‐SOX9 + XMU‐MP‐1. Cells in the logarithmic growth phase were harvested and injected subcutaneously into the right axillary/flank region of each mouse in a total volume of 100 μL containing 1 × 10^7^ cells.

Animals in the designated groups underwent XMU‐MP‐1 intervention according to the predefined experimental design, whereas the corresponding control groups were handled in parallel. Tumour growth monitoring was initiated 7 days after inoculation and performed twice weekly using callipers. Tumour volume was calculated using the formula: *V* = 0.5 × *L* × *W* ^2^. At 28 days after inoculation, mice were euthanized, and tumours were excised, photographed, and weighed. Tumour tissues were fixed in 10% neutral‐buffered formalin, embedded in paraffin, and sectioned at 4 μm thickness. For haematoxylin and eosin staining, sections were deparaffinized, rehydrated, stained with haematoxylin and eosin, dehydrated, cleared, and mounted for histopathological examination.

For immunohistochemistry, paraffin sections underwent antigen retrieval, followed by quenching of endogenous peroxidase activity with 3% H_2_O_2_ and blocking with 5% bovine serum albumin. Sections were incubated with primary antibodies against SOX9, Ki67, and CD68 (Bioss, bsm‐60634R). Immunohistochemical detection was performed using a PV‐9001 detection system. Immunoreactivity was visualized using DAB, counterstained with haematoxylin, and imaged under a light microscope. Staining intensity was quantified as average optical density using ImageJ software. For CD68 immunohistochemistry, three representative fields were analyzed for each tumour section, and the mean value from each tumour was used for group‐level comparison.

### 2.6 Statistical Analysis

Statistical analyses were performed using R and GraphPad Prism. Correlations in transcriptomic analyses were assessed using Pearson or Spearman tests as appropriate, and nonlinear trends along pseudotime were modelled using generalized additive models. Spatial enrichment and autocorrelation analyses were evaluated by permutation testing. In vitro data are presented as mean ± SD from at least three independent experiments and were analyzed using unpaired two‐tailed Student's *t*‐tests or one‐way ANOVA with appropriate post hoc tests, as applicable. Western blot band intensities and immunohistochemical average optical density values were analyzed using group‐level biological replicates. For immunohistochemical quantification, multiple microscopic fields from the same tumour were averaged before statistical comparison. Xenograft growth curves were analyzed using two‐way repeated‐measures ANOVA, and endpoint measurements were analyzed by one‐way ANOVA. A two‐sided *p* < 0.05 was considered statistically significant.

## 3 Results

### 3.1 Single‐Cell Trajectory Analysis Identifies Dynamic SOX9 Regulation

#### 3.1.1 Cellular Composition and Pseudotime Evolution

**FIGURE 1:**
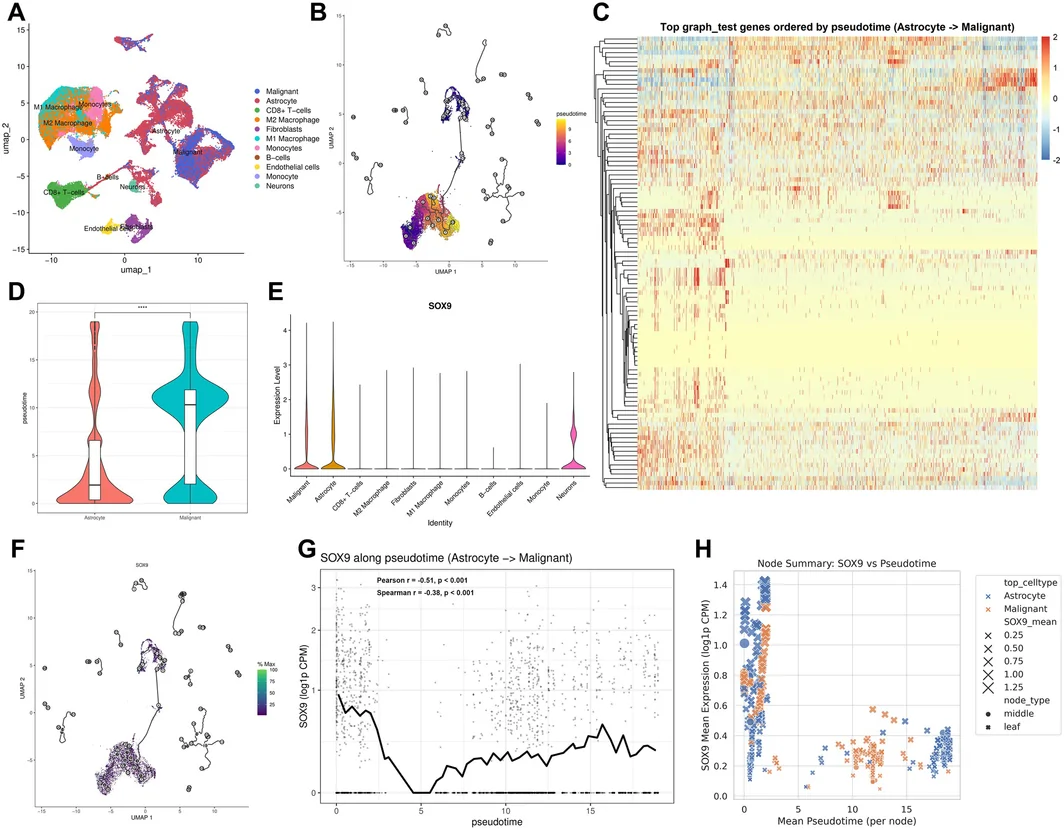
Single‐cell trajectory reconstruction and dynamic SOX9 expression in glioblastoma. (A) UMAP visualization of the major cell populations identified from the integrated single‐cell RNA‐seq dataset. (B) Monocle3 pseudotime trajectory showing the transition from astrocyte‐like states towards malignant states. (C) Heatmap of the top trajectory‐associated genes across pseudotime. (D) Pseudotime distributions of astrocytes and malignant cells. (E, F) SOX9 expression across major cell populations and along the pseudotime trajectory. (G, H) Correlation and node‐level summary of SOX9 expression with pseudotime.

Integrated single‐cell transcriptomic analysis revealed substantial cellular heterogeneity within glioma samples, including malignant cells, astrocytes, macrophages (M1/M2), T cells, B cells, monocytes, neurons, fibroblasts, and endothelial cells (Figure 1A). UMAP visualization provided a clear cellular framework for downstream trajectory inference.

Pseudotime analysis using Monocle3 delineated a continuous transition from astrocytes to malignant cells, indicating progressive conversion from glial to malignant states (Figure 1B). A pseudotime heatmap further illustrated dynamic changes in key gene modules along this trajectory (Figure 1C). Consistent with the inferred transition, violin plot analysis showed that astrocytes predominated at early pseudotime stages, whereas malignant cells were enriched at later stages (*p* < 0.001; Figure 1D).

#### 3.1.2 Temporal Dynamics of SOX9 Along Pseudotime

SOX9 expression was predominantly detected in astrocyte and malignant cell populations, with minimal expression in immune and stromal compartments (Figure 1E). Projection onto the pseudotime trajectory showed that SOX9 expression was highest at early astrocytic stages and progressively declined as cells transitioned towards malignant states (Figure 1F). Correlation analysis confirmed this downward trend (Pearson *r* = −0.507, *p* < 0.001; Spearman *r* = −0.38, *p* < 0.001; Figure 1G).

Further node‐level analysis showed that SOX9 expression was concentrated in early pseudotime nodes dominated by astrocytes and markedly reduced in nodes corresponding to malignant terminal states (Figure 1H), supporting a temporally restricted role for SOX9 during the astrocyte‐to‐malignant transition.

### 3.2 SOX9 Is Dynamically Coupled to Hippo‐Associated Programs

#### 3.2.1 SOX9 Inversely Associates With Hippo Kinase Activity

**FIGURE 2:**
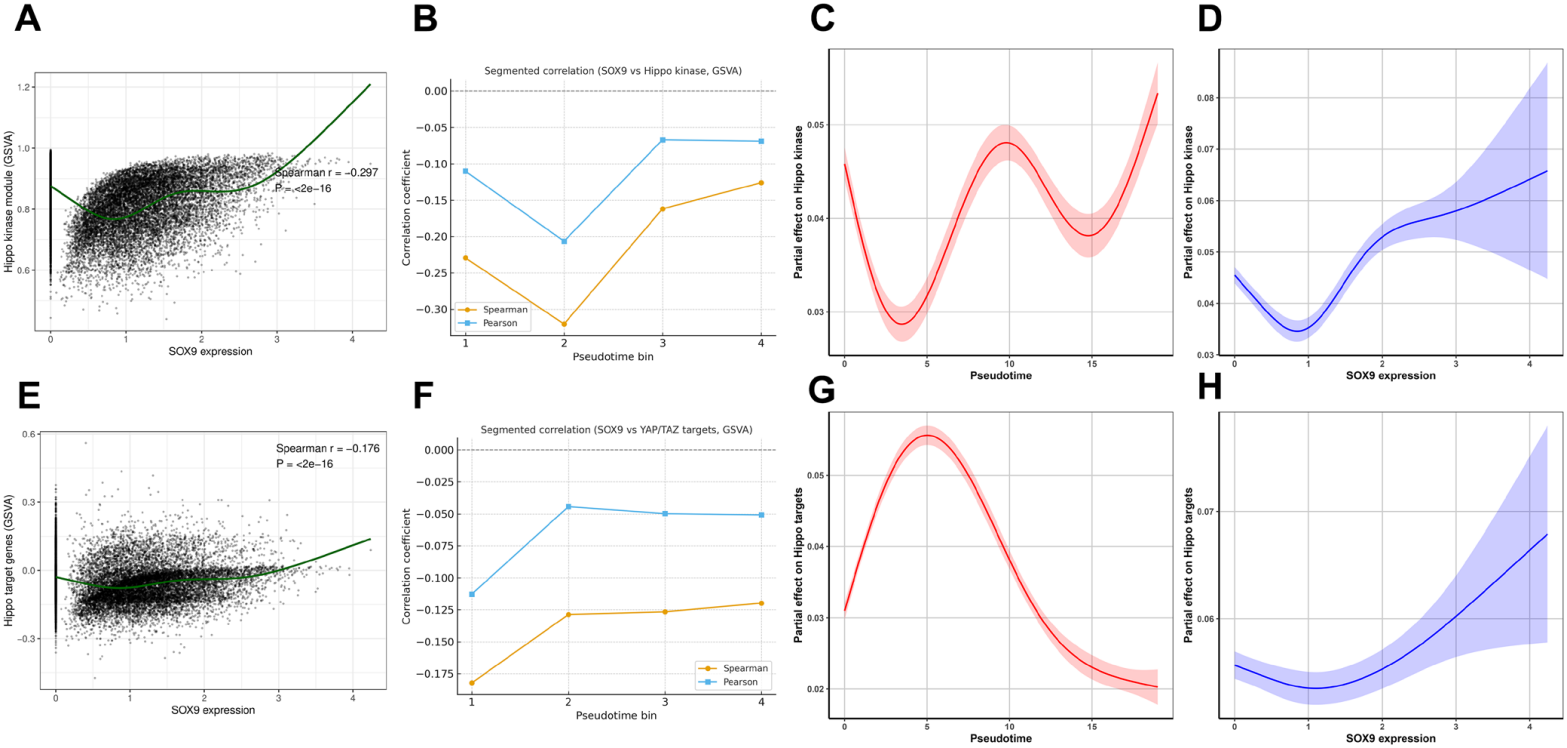
SOX9 is dynamically coupled with Hippo‐associated transcriptional programmes along pseudotime. (A) Global correlation between SOX9 expression and the upstream Hippo kinase module score. (B) Segmented correlation between SOX9 expression and Hippo kinase activity across pseudotime bins. (C, D) Generalized additive model analyses showing the effects of pseudotime and SOX9 on Hippo kinase module score. (E) Global correlation between SOX9 expression and the YAP/TAZ target gene module score. (F) Segmented correlation between SOX9 expression and YAP/TAZ target activity across pseudotime bins. (G, H) Generalized additive model analyses showing the effects of pseudotime and SOX9 on YAP/TAZ target gene module score.

To define the relationship between SOX9 and Hippo signalling, we quantified the activity of an upstream Hippo kinase module (MST1/2, LATS1/2, SAV1, MOB1A/B, NF2, WWC1) along pseudotime. GSVA scoring showed a negative correlation between SOX9 expression and kinase module activity (Spearman *r* = −0.297, *p* < 0.001; Figure 2A). Pseudotime‐stratified analysis indicated that this inverse association persisted across trajectory stages and was strongest during the early‐to‐intermediate phases (Figure 2B). GAM analysis further showed that both pseudotime and SOX9 significantly contributed to kinase module activity in a nonlinear manner (s (pseudotime), *p* < 0.001; s (SOX9), *p* < 0.001; Figure 2C,D), suggesting that the SOX9–Hippo relationship is phase‐dependent rather than uniformly monotonic.

#### 3.2.2 SOX9 Shows Phase‐Dependent Association With YAP/TAZ Targets

A canonical YAP/TAZ target gene set (CTGF, CYR61, ANKRD1, AMOTL2, AREG, SERPINE1, FOSL1, AXL, ITGA6, BIRC5) was then evaluated. SOX9 expression was negatively correlated with YAP/TAZ module activity (Spearman *r* = −0.176, *p* < 0.001; Figure 2E). This association was consistently maintained across pseudotime, particularly during early phases (Figure 2F). GAM analysis further supported independent nonlinear effects of SOX9 and pseudotime on target module activity (s (pseudotime), *p* < 0.001, s (SOX9), *p* < 0.001; Figure 2G,H).

### 3.3 Spatial Validation of SOX9–Malignant Coupling

**FIGURE 3:**
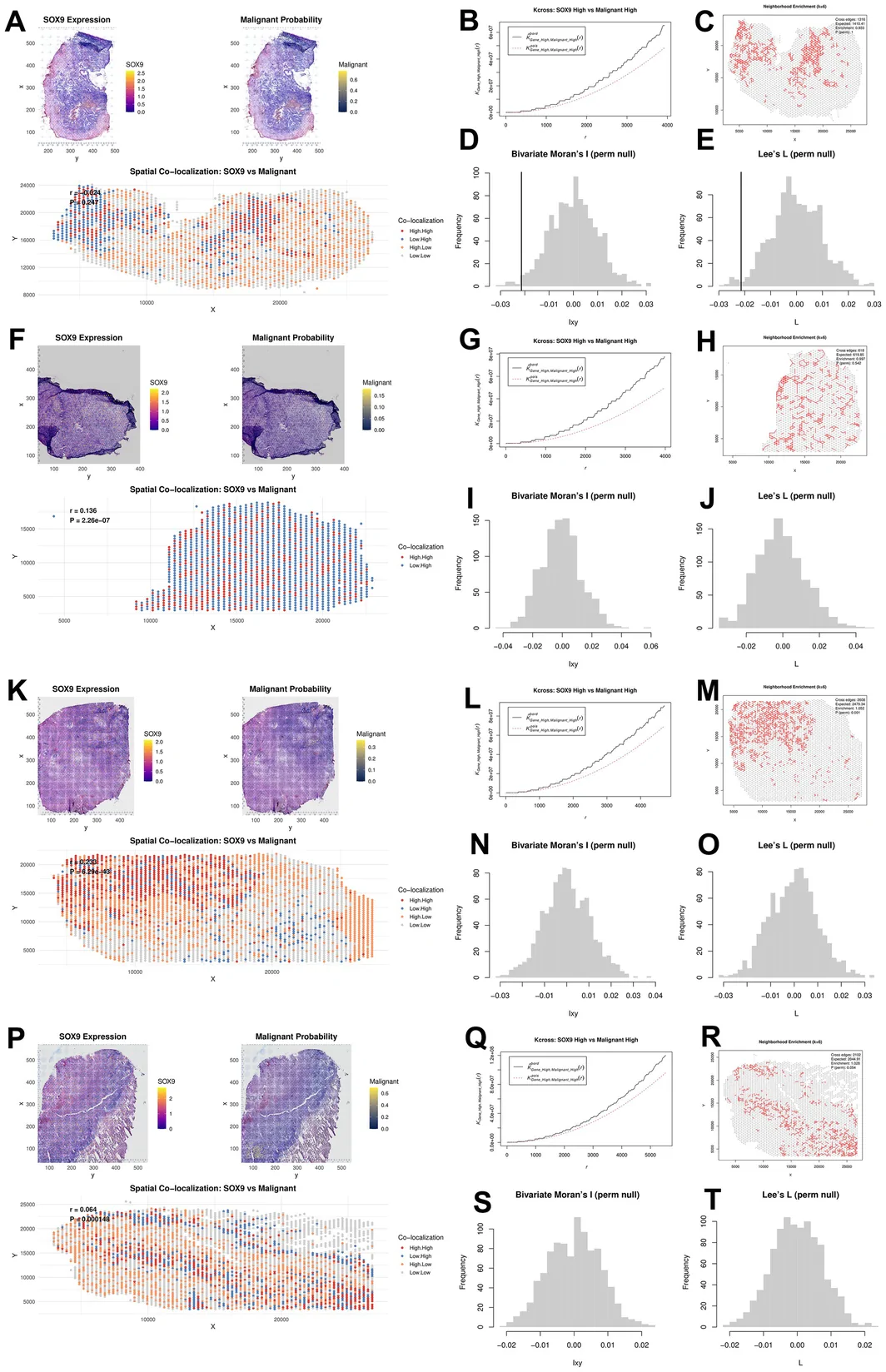
Spatial heterogeneity of SOX9–malignant coupling across glioblastoma regions. (A–E) Spatial mapping and co‐localization analyses of SOX9 expression and malignant cell probability in the tumour–normal interface sample. (F–J) Spatial mapping and co‐localization analyses in the pure tumour sample. (K–O) Spatial mapping and co‐localization analyses in the perivascular sample. (P–T) Spatial mapping and co‐localization analyses in the tumour–necrosis interface sample. For each region, panels include spatial expression/probability mapping, Kcross analysis, neighbourhood enrichment, bivariate Moran's *I*, and Lee's *L* statistics.

In the tumour–normal interface sample (GSM8013398), SOX9 was expressed at low‐to‐moderate levels and showed no significant global correlation with malignant cell probability (*r* = −0.024, *p* > 0.05; Figure 3A). Kcross analysis showed that SOX9‐high and malignant‐high spots exhibited clustering above Poisson expectation, suggesting a localized tendency towards spatial association (Figure 3B). However, neighbourhood enrichment analysis showed fewer cross‐type edges than expected (1316 vs. 1410.41; enrichment ratio = 0.933, *p* > 0.05; Figure 3C), and bivariate spatial autocorrelation analyses were not significant (Moran's *I* = −0.0214, *p* > 0.05; Lee's *L* = −0.0214, *p* > 0.05; Figure 3D,E), indicating no stable spatial coupling in the interface region.

In the pure tumour sample (GSM8013399), SOX9 expression broadly overlapped with malignant‐high regions. A significant positive global correlation was observed (*r* = 0.136, *p* < 0.001; Figure 3F). Kcross analysis further suggested localized clustering between SOX9‐high and malignant‐high spots above Poisson expectation (Figure 3G). However, neighbourhood enrichment was not significant (618 vs. 619.85; enrichment ratio = 0.997, *p* > 0.05; Figure 3H), and bivariate spatial autocorrelation analyses revealed statistically significant but modest positive correlations (Moran's *I* = 0.067, *p* < 0.001; Lee's *L* = 0.0671, *p* < 0.001; Figure 3I,J), suggesting global overlap without robust local coupling.

In the perivascular sample (GSM8013405), SOX9 expression was markedly upregulated and showed clear spatial concordance with malignant cell probability. Global correlation analysis revealed a significant positive association (*r* = 0.233, *p* < 0.001; Figure 3K). Kcross analysis further indicated a positive spatial association between SOX9‐high and malignant‐high regions, supporting spatial coupling between SOX9 and the malignant niche (Figure 3L). Neighbourhood enrichment analysis confirmed significant spatial coupling (2608 vs. 2479.34; enrichment ratio = 1.052, *p* < 0.001; Figure 3M). Consistently, bivariate spatial autocorrelation analyses demonstrated significant positive correlations (Moran's *I* = 0.2072, *p* < 0.001; Lee's *L* = 0.2073, *p* < 0.001; Figure 3N,O), indicating robust spatial coupling in perivascular regions.

In the tumour–necrosis interface sample (GSM8013410), SOX9 expression was diffusely distributed with focal enhancement. Global correlation analysis revealed a weak but statistically significant positive association with malignant cell probability (*r* = 0.064, *p* < 0.001; Figure 3P). Kcross analysis suggested a limited tendency towards positive spatial association between SOX9‐high and malignant‐high regions (Figure 3Q). Neighbourhood enrichment showed only a modest increase in cross‐type edges (2102 vs. 2044.91; enrichment ratio = 1.028, *p* > 0.05; Figure 3R), while bivariate spatial autocorrelation analyses remained weak and did not reach statistical significance (Moran's *I* = 0.0067, *p* > 0.05; Lee's *L* = 0.0042, *p* > 0.05; Figure 3S,T), suggesting limited and unstable spatial coupling at the necrotic margin.

### 3.4 SOX9‐Associated Phenotypic Changes In Vitro

**FIGURE 4:**
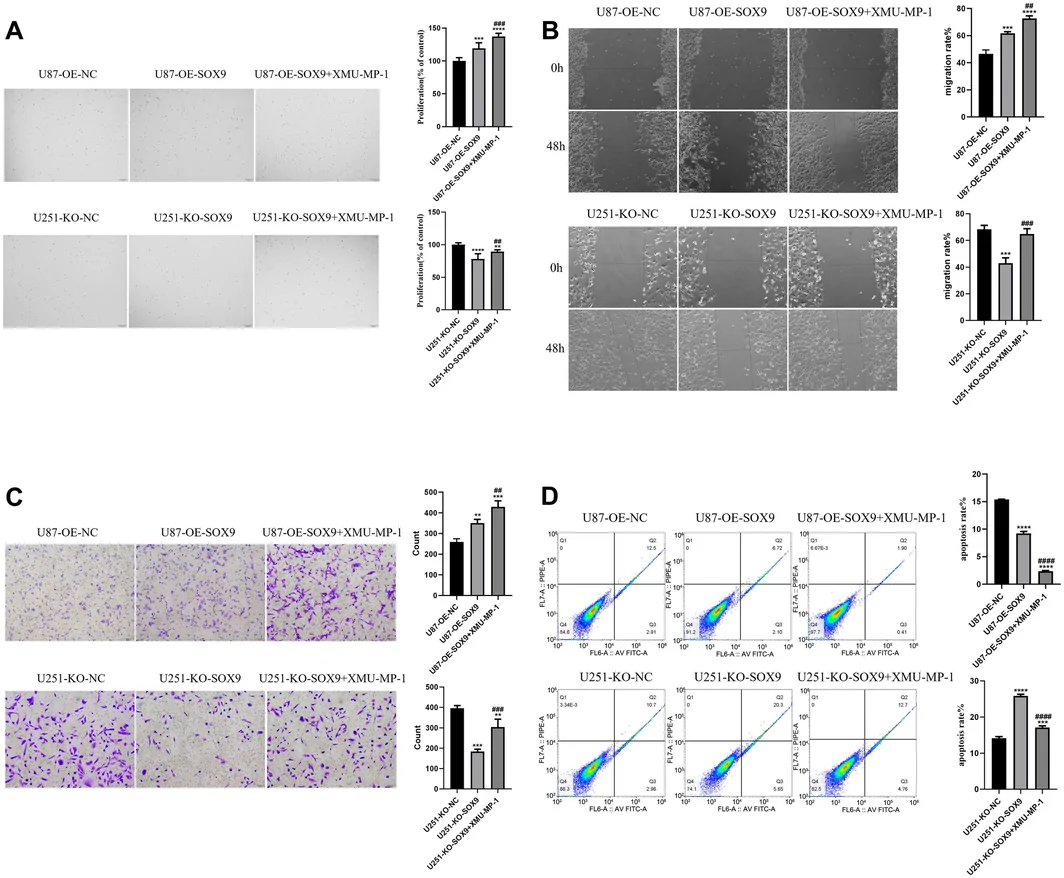
SOX9‐associated phenotypic changes in vitro. (A) Cell proliferation assessed by CCK‐8 assays in U87‐OE‐NC, U87‐OE‐SOX9, U251‐KO‐NC, U251‐KO‐SOX9, and XMU‐MP‐1‐treated groups. (B) Wound‐healing assays showing cell migration under the indicated conditions. (C) Transwell invasion assays showing invasive cell numbers under the indicated conditions. (D) Flow‐cytometric apoptosis analysis under the indicated conditions. Data are shown as mean ± SD from at least three independent experiments.

Cell proliferation assessed by CCK‐8 assays showed that SOX9 overexpression in U87 cells produced a modest but statistically significant increase in proliferation, whereas SOX9 knockout in U251 cells reduced proliferation (Figure 4A). XMU‐MP‐1 treatment partially rescued proliferation in SOX9‐deficient cells.

Wound‐healing assays showed increased migratory capacity in SOX9‐overexpressing U87 cells and impaired migration in SOX9‐knockout U251 cells (Figure 4B). XMU‐MP‐1 treatment partially rescued migration defects induced by SOX9 loss.

Transwell assays showed that SOX9 upregulation enhanced invasive capacity, whereas SOX9 depletion reduced transmembrane cell numbers (Figure 4C). These effects were partially reversed by XMU‐MP‐1.

Apoptosis assays showed that SOX9 overexpression decreased apoptosis in U87 cells, while SOX9 knockout increased apoptosis in U251 cells (Figure 4D). XMU‐MP‐1 attenuated apoptosis induced by SOX9 loss.

### 3.5 SOX9‐Associated Hippo–YAP/TAZ Pathway Changes

**FIGURE 5:**
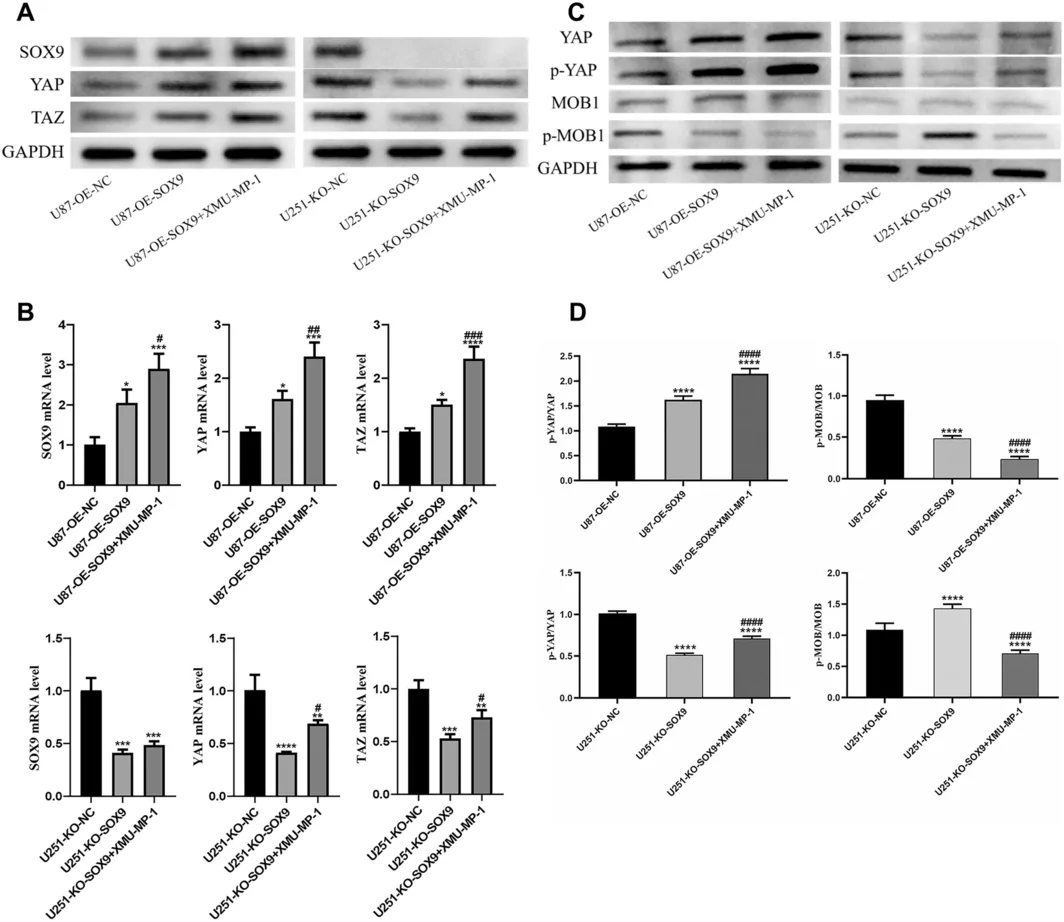
SOX9‐associated Hippo–YAP/TAZ pathway changes. (A) Representative Western blots showing SOX9, YAP, TAZ, and GAPDH protein expression in the indicated groups. (B) qRT‐PCR analysis of SOX9, YAP, and TAZ expression in U87‐OE‐NC, U87‐OE‐SOX9, U87‐OE‐SOX9 + XMU‐MP‐1, U251‐KO‐NC, U251‐KO‐SOX9, and U251‐KO‐SOX9 + XMU‐MP‐1 cells. (C) Representative Western blots showing YAP, p‐YAP, MOB1, p‐MOB1, and GAPDH in the indicated groups. (D) Quantification of phosphorylation‐related Hippo–YAP pathway readouts, including p‐YAP/YAP and p‐MOB1/MOB1 ratios. Data are shown as mean ± SD. In each three‐group comparison, asterisks indicate comparison with the NC group, and hash marks indicate comparison with the OE‐SOX9 or KO‐SOX9 group.*p < 0.05, **p < 0.01, ***p < 0.001, and ****p < 0.0001 versus the corresponding NC group; ^#^ *p* < 0.05, ^##^ *p* < 0.01, ^###^ *p* < 0.001, and ^####^ *p* < 0.0001 versus the corresponding SOX9‐manipulated group.

SOX9 overexpression in U87 cells increased YAP and TAZ expression at both the mRNA and protein levels, whereas SOX9 knockout in U251 cells reduced YAP/TAZ expression (Figure 5A,B; *p* < 0.05 or *p* < 0.001). In SOX9‐deficient cells, XMU‐MP‐1 treatment partially increased YAP/TAZ expression.

In phosphorylation‐related Western blot analysis, SOX9 gain and loss produced reciprocal changes in p‐YAP/YAP and p‐MOB1/MOB1 ratios. SOX9 overexpression in U87 cells increased the p‐YAP/YAP ratio and reduced the p‐MOB1/MOB1 ratio, whereas SOX9 knockout in U251 cells reduced the p‐YAP/YAP ratio and increased the p‐MOB1/MOB1 ratio. XMU‐MP‐1 treatment shifted both ratios towards the SOX9‐overexpression pattern, with increased p‐YAP/YAP and reduced p‐MOB1/MOB1 levels in the indicated groups (Figure 5C,D). Total MOB1 levels showed no marked group‐dependent change.

### 3.6 SOX9 Promotes Tumorigenicity In Vivo

**FIGURE 6:**
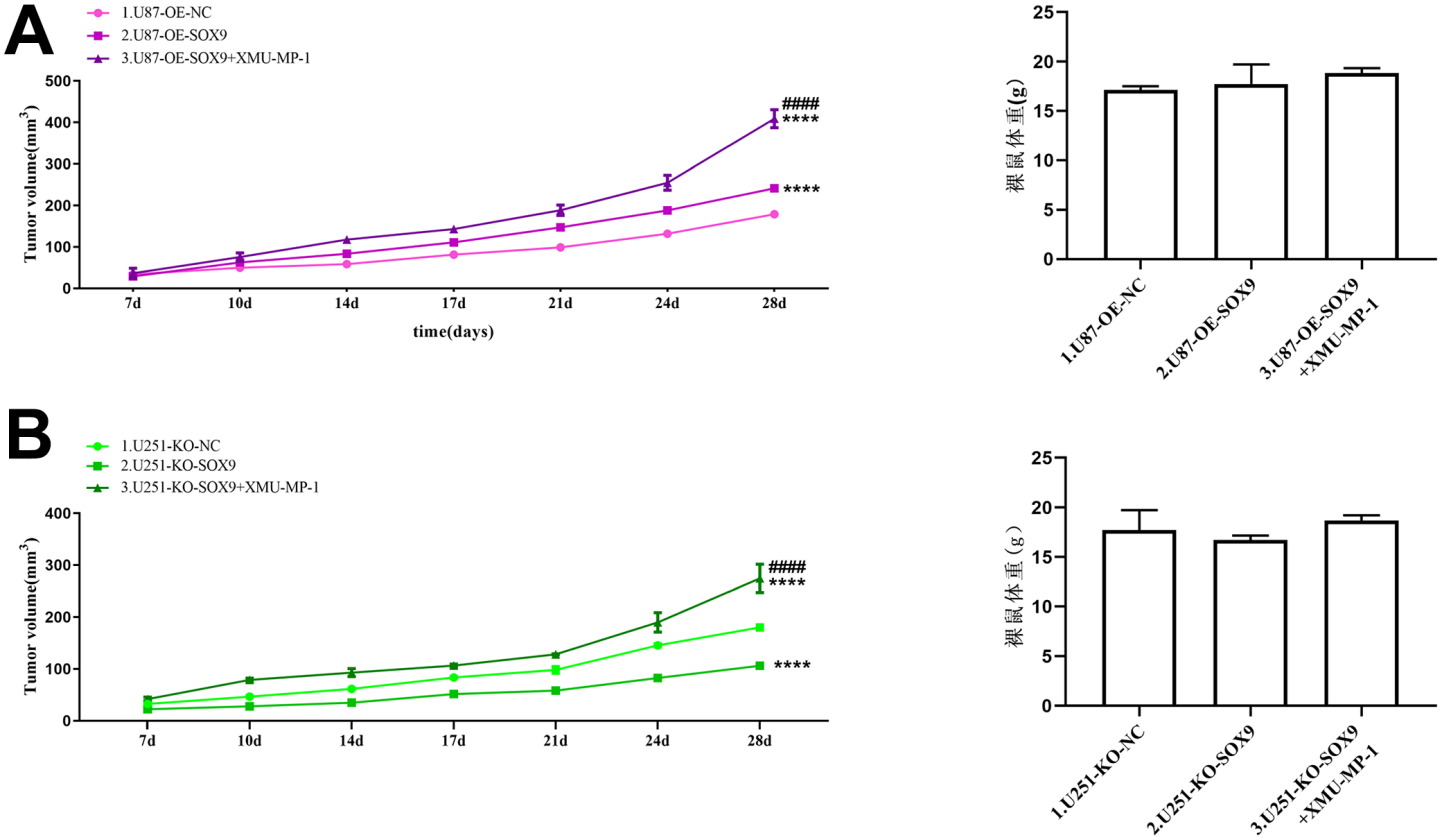
SOX9‐associated tumour growth changes in vivo. (A) Tumour growth curves of U87‐derived xenografts in the OE‐NC, OE‐SOX9, and OE‐SOX9 + XMU‐MP‐1 groups. (B) Tumour growth curves of U251‐derived xenografts in the KO‐NC, KO‐SOX9, and KO‐SOX9 + XMU‐MP‐1 groups.

In subcutaneous xenograft models (*n* = 3 per group), SOX9 overexpression significantly enhanced tumour growth in U87‐derived tumours, with differences becoming evident from day 14 onward and maintained through the experimental endpoint (Figure 6A; *p* < 0.0001 vs. OE‐NC). XMU‐MP‐1 treatment further augmented tumour expansion in SOX9‐overexpressing tumours (*p* < 0.0001 vs. OE‐SOX9 alone). Conversely, SOX9 knockout markedly suppressed tumour growth in U251 xenografts (*p* < 0.0001 vs. KO‐NC), and this inhibitory effect was partially reversed by XMU‐MP‐1 (*p* < 0.0001 vs. KO‐SOX9 alone; Figure 6B). These data showed that SOX9 modulation was associated with altered glioblastoma tumour growth in vivo, with partial reversal after XMU‐MP‐1 treatment.

### 3.7 Histopathological Changes Induced by SOX9 Modulation

**FIGURE 7:**
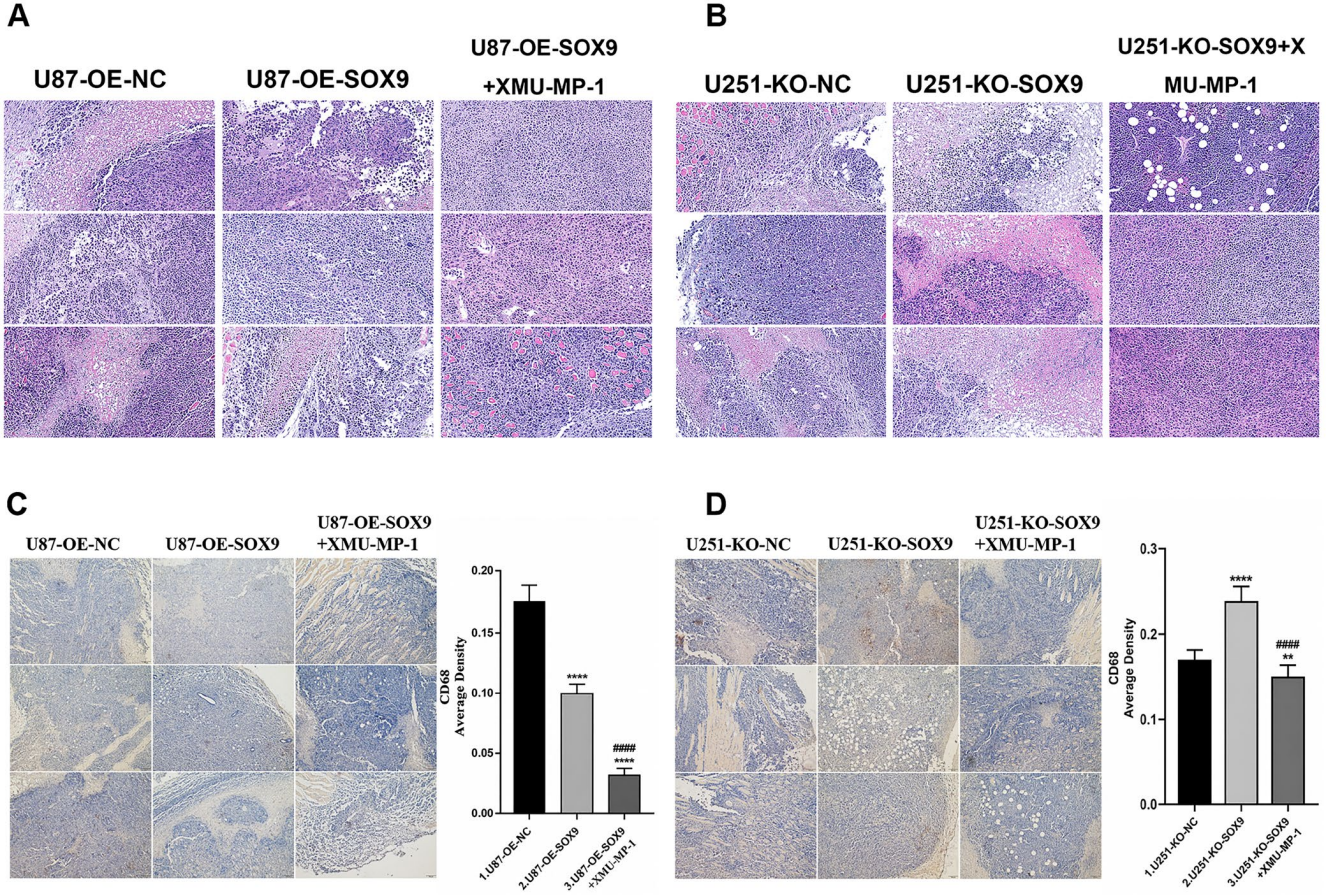
Histopathological and CD68 changes in glioblastoma xenografts after SOX9 modulation. (A) Representative H&E‐stained sections of U87‐derived xenografts from the OE‐NC, OE‐SOX9, and OE‐SOX9 + XMU‐MP‐1 groups. (B) Representative H&E‐stained sections of U251‐derived xenografts from the KO‐NC, KO‐SOX9, and KO‐SOX9 + XMU‐MP‐1 groups. (C) Representative CD68 immunohistochemical staining and quantitative analysis of CD68 average density in U87‐derived xenografts. (D) Representative CD68 immunohistochemical staining and quantitative analysis of CD68 average density in U251‐derived xenografts. Data are presented as mean ± SD. For CD68 quantification, three representative fields from each tumour section were analyzed, and the mean value from each tumour was used for group‐level comparison. *p < 0.05, **p < 0.01, ***p < 0.001, and ****p < 0.0001 versus the corresponding NC group; ^#^ *p* < 0.05, ^##^ *p* < 0.01, ^###^ *p* < 0.001, and ^####^ *p* < 0.0001 versus the corresponding SOX9‐manipulated group.

Haematoxylin and eosin staining revealed distinct pathological differences across experimental groups (Figure 7A,B). In U87 xenografts, SOX9 overexpression was associated with increased tumour necrosis and nuclear atypia compared with controls, and these alterations were further aggravated by XMU‐MP‐1 treatment. Conversely, in U251‐derived tumours, SOX9 knockout was associated with reduced necrotic changes and less evident histological atypia relative to controls, whereas XMU‐MP‐1 administration partially reversed these changes.

CD68 average density was reduced in U87‐OE‐SOX9 tumours compared with U87‐OE‐NC tumours, with a further decrease in the U87‐OE‐SOX9 + XMU‐MP‐1 group (Figure 7C). In U251‐derived tumours, CD68 average density was increased in the U251‐KO‐SOX9 group compared with U251‐KO‐NC tumours and was reduced after XMU‐MP‐1 treatment (Figure 7D).

### 3.8 Immunohistochemical Validation In Vivo

**FIGURE 8:**
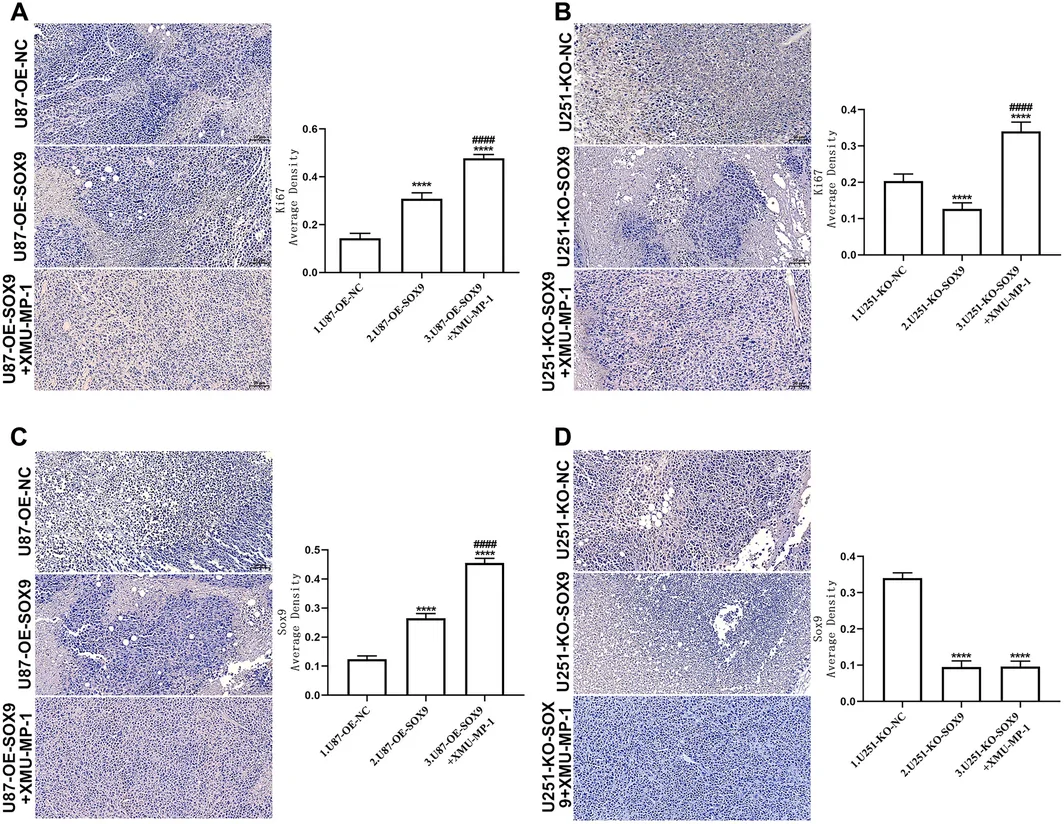
Immunohistochemical validation of SOX9 expression and proliferative activity in glioblastoma xenografts. (A, B) Representative Ki67 staining and quantification of average optical density (AOD) in U87 and U251 xenografts under the indicated conditions. (C, D) Representative SOX9 staining and AOD quantification in U87 and U251 xenografts under the indicated conditions. Data are presented as mean ± SEM (*n* = 3 mice per group). ****p < 0.0001 vs. corresponding control group; ^####^ *p* < 0.0001 vs. corresponding SOX9‐modulated group. Scale bars, 50 μm.

Ki67 immunohistochemistry demonstrated significantly increased proliferative activity in U87‐OE‐SOX9 tumours compared with controls, with further enhancement following XMU‐MP‐1 treatment (Figure 8A). In contrast, Ki67 positivity was reduced in U251‐KO‐SOX9 tumours relative to controls and was increased in the U251‐KO‐SOX9 + XMU‐MP‐1 group compared with KO‐SOX9 alone (Figure 8B).

SOX9 immunohistochemical staining confirmed effective modulation of SOX9 expression in vivo. Strong nuclear SOX9 staining was observed in U87‐OE‐SOX9 tumours and was further increased following XMU‐MP‐1 treatment (Figure 8C). In U251‐derived xenografts, SOX9 expression was markedly reduced in both the KO‐SOX9 and KO‐SOX9 + XMU‐MP‐1 groups compared with controls (Figure 8D).

## 4 Discussion

The present study provides three principal advances in the understanding of SOX9‐driven glioblastoma progression. First, it identifies SOX9 as a temporally restricted regulator during the acquisition of malignant stem‐like traits, rather than a constitutively required stemness‐maintaining factor throughout tumour evolution. Second, it demonstrates that SOX9–Hippo–YAP/TAZ coupling is spatially heterogeneous across the glioblastoma microenvironment, with the strongest and most stable association observed in the perivascular niche. Third, it supports an “early priming–late decoupling” model in which SOX9 is linked to early malignant reprogramming, whereas downstream programmes become progressively less dependent on SOX9 once malignant states are established.

A central finding of this study is that SOX9 does not behave as a uniformly sustained driver across the entire glioblastoma trajectory. Instead, single‐cell pseudotime analysis showed that SOX9 expression peaked during the astrocyte‐to‐malignant transition and declined after malignant states became stabilized. This temporal pattern suggests that SOX9 functions primarily during a defined window of state conversion. In biological terms, SOX9 appears to act less as a permanent executor of stemness and more as a transition‐enabling regulator that appears to lower the threshold for malignant reprogramming [10, 11]. This interpretation also explains why stemness‐associated phenotypes can remain active in advanced tumour states despite reduced SOX9 expression.

Mechanistically, this temporally restricted role is supported by the behaviour of Hippo‐associated programmes along pseudotime. SOX9 showed an inverse association with the upstream Hippo kinase module, whereas its relationship with YAP/TAZ target activity was phase‐dependent rather than uniformly monotonic [12]. Together with the gain‐ and loss‐of‐function experiments, these findings indicate that SOX9 exerts its most critical regulatory effect during the early transition window, when changes in Hippo kinase‐related activity may be accompanied by engagement of YAP/TAZ‐associated transcriptional output [13]. Phosphorylation‐related analyses showed concordant changes in YAP, p‐YAP, and p‐MOB1 after SOX9 modulation and XMU‐MP‐1 treatment, extending the transcriptomic association to protein‐level Hippo–YAP pathway readouts [13]. Once this downstream circuitry is established, malignant and stemness‐related transcriptional programmes can be maintained despite declining SOX9 expression [4]. On this basis, an “early priming–late decoupling” model is proposed: SOX9 acts early to initiate Hippo–YAP/TAZ‐linked malignant reprogramming, whereas later tumour states increasingly rely on consolidated downstream networks and become less dependent on continued high SOX9 expression.

The spatial transcriptomic findings extend this model from the temporal dimension to the tissue level. SOX9–malignant coupling was not spatially uniform across glioblastoma regions [14]. It was weak or unstable at the tumour–normal interface and necrotic margin, modest within the pure tumour core, and most robust in the perivascular niche. This regional pattern is biologically meaningful [15]. The perivascular niche is a recognized reservoir for stem‐like glioblastoma cells and provides a microenvironment enriched in vascular, hypoxic, and paracrine signals that support cellular plasticity [16, 17, 18]. The present findings therefore suggest that SOX9‐dependent reprogramming is not only time‐restricted but also niche‐conditioned, with perivascular regions representing the most permissive anatomical context for effective SOX9–Hippo–YAP/TAZ coupling. In contrast, in other regions, downstream malignant programmes may be sustained through alternative inputs or only partial coupling, resulting in weaker spatial coherence.

These findings refine, rather than simply repeat, previous views of SOX9 in glioblastoma. Earlier studies, largely based on bulk transcriptomic profiling, immunohistochemistry, or static functional analyses, have generally interpreted SOX9 as a continuously active stemness‐promoting factor [19, 20, 21]. Such studies established the oncogenic relevance of SOX9, but lacked the temporal and spatial resolution needed to distinguish early transitional activation from persistent downstream dependence. The present study adds this missing dimension. It does not dispute the importance of SOX9 in glioblastoma; instead, it repositions SOX9 within a dynamic regulatory framework, in which its function is strongest during early malignant conversion and most effectively stabilized within specific microenvironmental niches. Similarly, while the Hippo–YAP/TAZ axis has long been implicated in glioblastoma plasticity, the current data suggest that SOX9 is linked to this pathway in a stage‐dependent and region‐specific manner, rather than through a simple static linear relationship.

The study also has several strengths. By integrating single‐cell transcriptomics, spatial transcriptomics, in vitro functional assays, phosphorylation‐related Western blotting, CD68 immunohistochemistry, and in vivo validation, it connects cell‐state transition, tissue context, and biological consequence within a unified framework [22]. This design allows SOX9 to be interpreted not merely as a differentially expressed marker, but as a regulator positioned at the intersection of temporal transition, spatial niche selection, and malignant phenotype acquisition. CD68 immunohistochemistry also refined the interpretation of xenograft histopathology by distinguishing general H&E‐based morphological changes from CD68‐positive myeloid/macrophage‐like cell accumulation. At the same time, several limitations should be acknowledged. The transcriptomic analyses were based on public datasets with limited cohort size and subtype diversity. Functional validation relied primarily on established cell lines and subcutaneous xenograft models rather than patient‐derived or orthotopic systems [23]. Although p‐MOB1 and p‐YAP were assessed, YAP/TAZ nuclear localization, inhibitor dose–response testing, and orthogonal pharmacological validation using a second Hippo‐pathway inhibitor were not performed. Additional glioma cell‐line models were also not included in the functional assays [24]. These issues limit mechanistic depth and should be addressed in future work.

The present findings have both biological and translational implications. Biologically, they suggest that SOX9 should be considered a temporally restricted state‐switch regulator rather than a static stemness marker [25]. Spatially, they indicate that the SOX9–Hippo–YAP/TAZ axis operates in a niche‐dependent manner and is most effectively engaged in the perivascular compartment [26]. Translationally, this distinction is important. If SOX9 acts predominantly during an early transition window, therapeutic strategies directed against SOX9 may be most effective before malignant programmes become fully consolidated. Conversely, in later‐stage tumours, inhibition of SOX9 alone may be insufficient, because downstream transcriptional networks and niche‐derived signals may already sustain the malignant state independently. These observations support the concept of stage‐specific and niche‐aware therapeutic intervention, particularly in approaches targeting the SOX9/Hippo/YAP–TAZ regulatory axis [27].

## 5 Conclusions

The present study identifies a temporally restricted role for SOX9 in glioblastoma stemness acquisition, demonstrates that SOX9–Hippo–YAP/TAZ coupling is region‐specific within the tumour microenvironment, and proposes an “early priming–late decoupling” model for malignant state transition. This framework refines the current understanding of SOX9 biology in glioblastoma and provides a conceptual basis for future stage‐aware and microenvironment‐directed therapeutic strategies.

## Author Contributions

**Nuerzhati Wusiman:** conceptualization, project administration. **Minghao Lian:** data curation, investigation, project administration, methodology. **Gaocai Zhang:** investigation, methodology, project administration, data curation. **Chunyu Song:** methodology, investigation, project administration, data curation. **Xiaoqin Chen:** project administration, conceptualization. **Xiahela Xiaokaiti:** conceptualization, project administration. **Maimaitili Mijiti:** investigation, funding acquisition, project administration, writing – original draft. **Hua Shi:** conceptualization, methodology, project administration, writing – review and editing, visualization, software. **Aierpati Maimaiti:** investigation, data curation, project administration, methodology. **Guohua Zhu:** conceptualization, project administration. **Jufan Wang:** methodology, investigation, project administration, data curation. **Yongtao Zhang:** investigation, methodology, project administration, data curation. **Dangmurenjiafu Geng:** conceptualization, project administration. **Yunbo Wang:** methodology, data curation, investigation, project administration. **Xiangrui Kong:** conceptualization, project administration. **Yandong Li:** project administration, funding acquisition, writing – original draft.

## Funding

This work was supported by the Natural Science Foundation of Xinjiang Uygur Autonomous Region, China (2023D01C98) and the Youth Scientific Research Start‐up Special Fund of the First Affiliated Hospital of Xinjiang Medical University (2022YFY‐QKQN‐62).

## Ethics Statement

All animal experiments were approved by the Institutional Animal Care and Use Committee (IACUC) of Obio Technology (Shanghai) Corp. Ltd. (approval no. 171). All procedures were conducted in accordance with the Guidelines for the Welfare and Ethical Review of Laboratory Animals (GB/T 35892‐018) and adhered to the principles of Replacement, Reduction, and Refinement (3Rs). Humane endpoints were observed throughout the study, and humane euthanasia was performed at the end of the experiments. All procedures were carried out by qualified personnel, and all animals were obtained from licensed institutions authorized for laboratory animal production.

## Conflicts of Interest

The authors declare no conflicts of interest.

## Data Availability

The datasets analyzed during the current study are publicly available in the Gene Expression Omnibus (GEO) repository. The single‐cell RNA sequencing dataset is available under accession number GSE182109, and the spatial transcriptomic dataset is available under accession number GSE253080. Processed data and analysis scripts supporting the findings of this study are available from the corresponding authors upon reasonable request.
